# Orofacial Manifestation and Dental Management of Sickle Cell Disease: A Scoping Review

**DOI:** 10.1155/2021/5556708

**Published:** 2021-10-22

**Authors:** Mayank kakkar, Kristen Holderle, Megha Sheth, Szilvia Arany, Leslie Schiff, Adela Planerova

**Affiliations:** ^1^Department of General Dentistry, Eastman Institute for Oral Health, University of Rochester, Rochester, NY, USA; ^2^Department of Psychiatry and Pediatrics, University of Rochester Medical Center, Rochester, NY, USA

## Abstract

**Aims:**

Sickle cell disease (SCD) is an upcoming global health problem with rapid progress in therapy especially since 2017. However, systematic reviews found no clinical trials on the dental treatment of sickle cell disease (SCD). This article aims to outline the oral features of the sickle disease and discuss oral management strategies that can serve as guidelines for dental professionals. *Material and Methods*. A comprehensive literature review was conducted using PubMed, Google Scholar, and Web of Science. The search strategies were developed to cover publications from January 2010 to March 2020. With the help of keywords, multiple abstracts were identified. These abstracts were further reviewed, which included the information about the SCD manifestation, particularly about the oral health features. Based on all these articles and clinical experience, a narrative review was constructed, which summarizes all the aspects of the oral manifestation in people with SCD.

**Results:**

The results of this study demonstrate that there is distinct evidence available, indicating the developmental enamel defect leading to hypoplasia and increasing susceptibility to dental caries. Another important result of this review found that people with SCD have a vaso-occlusive crisis in the microcirculation in the dental pulp leading to symptomatic and asymptomatic pulpal necrosis without any signs of odontogenic pathology in an apparently healthy tooth. The study also found that early detection, intervention, and prevention are crucial for improving oral health care, and involving a multidisciplinary approach plays an important role in managing people with SCD.

**Conclusion:**

Patients with sickle cell disease have chronic overall health problems. The hematological disorder becomes their main concern and impaired oral health becomes secondary, increasing the risk for dental caries at the most. This paper broadly describes the oral manifestations of SCD, additionally; this paper also provides recommendations for better dental management of patients with SCD. Patients with SCD are often misjudged and, due to lack of knowledge and guidelines, dental providers are not able to provide adequate care. This paper attempts to highlight the essential measures to provide better dental care.

## 1. Introduction

Sickle cell disease (SCD) is a group of disorders that cause red blood cells to become misshapen and break down prematurely. In SCD, there is an abnormality of the hemoglobin that carries oxygen to cells throughout the body [1]. The abnormal hemoglobin, known as hemoglobin S, has a lower functional capacity and causes multiple systemic complications. Hemoglobin S distorts the shape of the red blood cell into a sickle or crescent shape, giving the disease its name [1].

SCD is one of the most common inherited blood disorders in the United States. According to the Centers for Disease Control and Prevention (CDC), it is estimated that over 100,000 Americans have SCD [2]. Though the exact number of people living with SCD is unknown, it has been estimated that 1 of every 365 African American babies is born with SCD while 1 of every 13 has the sickle cell trait [2]. The U.S. incidence estimate for sickle cell trait (based on information provided by 13 states) was 73.1 cases per 1,000 black newborns, 3.1 cases per 1,000 white newborns, and 2.2 cases per 1,000 Asian or Pacific Islander newborns. The incidence estimate for Hispanic ethnicity (within 13 states) was 6.9 cases per 1,000 Hispanic newborns [2]. Combinedly almost 90% of the world's SCD population collectively lives in three countries, that is, Nigeria, India, and the Democratic Republic of Congo [3, 4]. According to the World Health Organization (WHO), it is estimated that approximately 5% of the entire world population caries trait genes for the hemoglobin disorders, out of which mainly, sickle cell disease and thalassemia are more prevalent [5]. It has been approximated that the number of children born with sickle cell disease is expected to grow by nearly 30% from 2010 to 2050 [6]. The great number of the newborn with the SCD occurs in lower- and middle-income countries and due to lack of the early diagnosis and treatment, most of the affected die in the first few years of life, with reported excess mortality reaching up to 92% [7].

The structure of normal adult hemoglobin (Hb-A) molecules contains four polypeptide chains, two alpha units and two beta units [8]. Each chain includes one heme group, which acts as a binding site for the oxygen molecule. Both chains have distinct sequences of amino acids, which fold up to form different three-dimensional structures. The four chains are bound together by noncovalent interactions.

In SCD, a point mutation changes glutamic acid to valine in the hemoglobin beta (*β*) chain. This type of abnormal hemoglobin is known as hemoglobin S (Hb-S), and it causes red blood cells to become stiff and abnormally shaped. Instead of having its normal, round disk shape, the red blood cell is distorted into crescent or sickle shape.

In SCD, the life span of red blood cells is severely diminished from the usual 90–120 days to about 10 days [8]. Due to atypical hemoglobin and their sickle shape, red blood cells break down prematurely in the spleen, causing fewer overall red blood cells and leading to sickle cell anemia and hyperbilirubinemia. Since hemoglobin in the RBC is the main molecule that delivers oxygen to all the cells throughout the body, sickle cell anemia results in multiple symptoms of oxygen deficit, including fatigue, irritability, dizziness, lightheadedness, tachycardia, and shortness of breath. Furthermore, the rapid breakdown of RBC, hemolytic anemia, may also cause yellowing of the eyes and skin, known as jaundice. Oral health consequences of hemolytic anemia are generalized paleness of the oral mucosa and pain due to vaso-occlusive crisis within the microcirculation of the dental pulp.

## 2. Materials and Methods

### 2.1. Selection Criteria

In this study, literature related to sickle cell disease and oral symptoms was reviewed. The literature describing SCD, oral manifestation, and dental management includes controlled clinical studies, retrospective studies, and experimental studies and review.

### 2.2. Search Strategy

The descriptive search including PubMed and Medline (1946–present), CINAHL, Cochrane Central Register of Controlled Trials, Embase, Web of Science, Google Scholar, the US National Institutes of Health Trials Registry, WHO Library, IndMED, LILACS, and African Index Medicus until October 2020, with no language filter. Additional dental organization websites were searched, including the American Dental Association, to identify articles and statistics that examined an association between the sickle cell disease and oral manifestations. Details of the search strategy are provided in Table 1.

### 2.3. Search Terms

The search terms are as follows: Sickle cell disease, Oral Health, and Dental Symptoms.

#### 2.3.1. Inclusion Criteria

The literature included in this study was based on the following inclusion criteria:

1. Studies discussing sickle cell disease and dental/oral symptoms
2. Studies reporting sickle cell disease and oral manifestations
3. Studies on sickle cell disease and dental management

#### 2.3.2. Exclusion Criteria

The literature eligible for inclusion in this study was based on the following exclusion criteria:

1. Literature discussing anemia
2. Literature considering medical management
3. Literature on language other than English

### 2.4. Data Collection and Analysis

#### 2.4.1. Selection of Studies and Data Extraction

**Figure 1:**
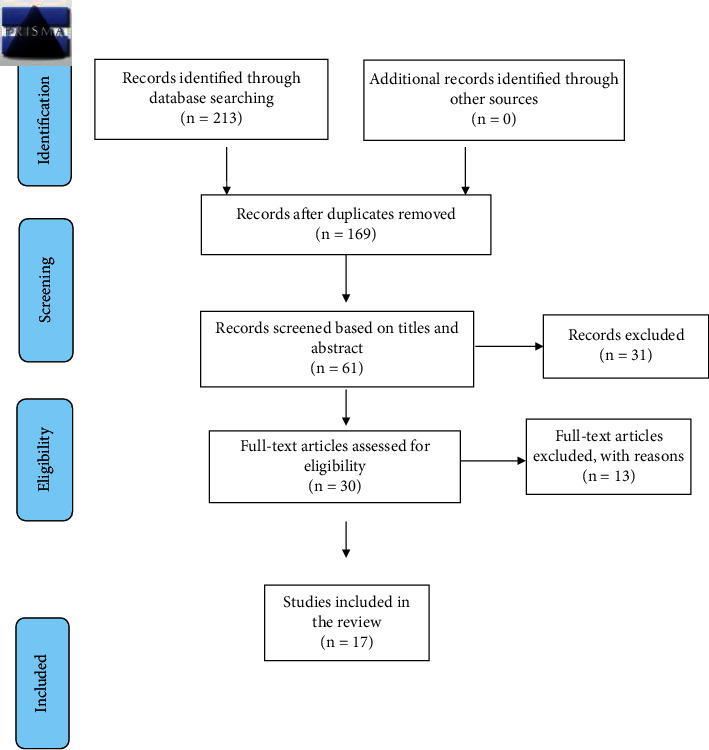
PRISMA 2009 flow diagram.

The articles were evaluated for their relevance based on the titles and abstracts. Further validity of the articles was done by obtaining the full text of the possible relevant studies that met the inclusion criteria. All the articles were reviewed by the reviewers. The studies assessed by MK and deemed eligible were checked by SA and AP for methodological quality and inclusion criteria. All disagreements were resolved verbally, with strict adherence to the predetermined inclusion criteria (refer to Figure 1).

## 3. Results

A narrative review was constructed reporting items with the question focused on “What the different oral manifestation observed in the patients with the SCD and how these patients are can better be managed.” *Population (P):* patients with SCD; *intervention (I):* common dental procedures at a clinical setting; *control (C):* no treatment, healthy controls; and *outcome measure (O):* dental management approaches. The aim of this article is to outline the oral features of SCD and discuss oral management strategies that can serve as guidelines for dental professionals.

### 3.1. Summary of the Intraoral and Dental Manifestations

#### 3.1.1. Oral Mucosa and Tongue

The most common intraoral manifestation of SCD is mucosal pallor and jaundice. This is caused by premature breakdown of RBCs in the spleen and the low number of available RBCs in the blood vessels leading to hemolytic anemia and hyperbilirubinemia [9, 10]. Due to the low blood oxygen, the color of the skin turns pale. This also can be observed in the intraoral buccal and labial mucosa, as well as the gingiva [9].

#### 3.1.2. Enamel and Dentin

There have been conflicting research results about the effect of SCD on teeth. A microradiography study of the dental tissues in SCD patients revealed diffused hypomineralized zones in tooth enamel. The study also found unusual inclusions in the lumens of the dentinal tubules and pulp chambers were found to contain denticle-like calcified bodies [11]. Many studies have reported enamel hypoplasia, dentin hypoplasia, and delayed tooth eruption in SCD patients [12]. There has been no distinct, evidence-based research demonstrating an association between SCD and greater risk of caries. However, there are several studies indicating that developmental enamel defects such as hypoplasia are postulated to have increased susceptibility to dental caries [12–14]. Defective enamel sites (hypoplasia or hypocalcification) may provide a suitable local environment for adhesion and colonization of cariogenic bacteria, and bacteria may be retained at the base of defects in contact with exposed dentin, enabling dental caries to develop more rapidly [14, 15]. Some studies have reported that patients with SCD are less susceptible to early childhood caries. Fakuda et al. concluded that that long-term use of penicillin prophylaxis in SCD patients may prevent the acquisition of Mutans Streptococci, resulting in significantly lower caries rates in this population. This benefit occurs only during active administration of the drug, however, and only delays the acquisition of Mutans Streptococci [16].

#### 3.1.3. Dental Pulp

The primary reason people with SCD visit dental providers is extreme pain and sensitivity. One way to explain this pain is due to caries approaching the pulp, resulting in inflammation of the pulp, a condition known as pulpitis. Another effect of SCD on the dental pulp is vaso-occlusive crisis, when obstruction of the microcirculation in the pulp produces symptomatic and asymptomatic pulpal necrosis without any signs of odontogenic pathology in an apparently healthy tooth [17–19].

Furthermore, arbitrated blood supply may cause blood clots within the blood vessel, commonly known as blood thrombosis, which can result in calcified pulp stones in the pulp chamber [10, 11].

#### 3.1.4. Mental Nerve Neuropathy: “Numb Chin Syndrome”

Vasculo-occlusion (VOC) in the maxillofacial region can also occur in the narrow canals of major nerves supplying the maxilla and the mandible causing loss of sensation and neuropathy. Because they pass through the narrow foramina and boney canals, the mental nerve and inferior alveolar nerve are the two major nerves that are vulnerable to VOCs. This infarction of the blood supply to the nerves can cause loss of sensation and persistent anesthesia to the lower lip and chin, which can last up to 24 months.

The first to describe mental nerve neuropathy as a result of SCD was Konotey-Ahulu in 1980. He found that 4% of patients had moderate-to-severe pain in the mandible during a sickle cell crisis, with many developing burning sensations and numbness in the lower lip along the path of the mental nerve. That recovery of sensation could take months [19]. Another case report was of a 40-year-old black man who described that his right mandibular first premolar, canine, and incisors “felt like wooden blocks.” A needle prick test was performed, and it was determined that the patient had profound anesthesia in the regions supplied by the mental nerve. A radiograph of the right mental nerve showed a 2 × 1 cm ovoid radiolucency, which was deciphered to be decreased trabeculation or an acute bony infarct of the mandible as a result of his sickle cell crisis. This lesion in the mandible was similar to other lesions found in the patient's pelvis and both right and left femoral heads. The pain eventually disappeared, although there was still numbness in the lower lip. The patient was followed for 12 months, with no changes in radiographic findings of radiolucency and loss of sensation in the lower lip [10, 20].

#### 3.1.5. Alveolar Bone and Radiographic Manifestation

Radiographic features in SCD have multiple causes. First and foremost is bone marrow hypertrophy and erythroblastic hyperplasia due to increased numbers of sickle cells and their premature destruction, causing low numbers of RBCs. Consequences of this are changes in the trabecular pattern of the bone, including loss of fine trabeculae and formation of large bone marrow spaces [9, 10, 12]. Thus, dental radiographs may appear to have distended medullary spaces and diminished trabeculation. There may be thinning of the cortical plate, and the inferior border of mandible may appear irregular and dissipated on the radiographic [10].

Another important feature of the SCD patient is developmental enamel hypomineralization and hypoplasia, which can affect enamel translucency and may be seen radiographically.

Maxillary sinus opacification may be observed in patients with SCD due to bone marrow hyperplasia of the maxillary sinus [10, 21–23].

There are many challenges when working with patients with SCD. We have reviewed these challenges in more detail in Table 2.

### 3.2. General Recommendations for Oral Health Management in Dental Practice

#### 3.2.1. Early Intervention

Patients with SCD are often seen in the emergency department due to severe pain. Likewise, they frequently present to dental clinics for emergency appointments rather than preventive care. Thus, by the time they come to a dental office, their oral health is quite deteriorated. Preventive dental therapy is ideal for sickle cell disease patient. The goal of the pediatric dentist is to improve and maintain excellent oral health in order to decrease the possibility of various oral infections [24]. Treatment should never be initiated during a crisis unless it is inevitable as in emergency situations.

Hence, it is important to establish routine dental [1] visits and comprehensive care from the beginning. It is crucial that patients are educated about good oral hygiene and encouraged to have periodic oral health screening and prophy at least every 6 months [25]. Individuals with SCD should be encouraged by their medical providers to seek regular dental care.

It is critical to maintain a multidisciplinary and collaborative approach to health care management, including the primary care physician, hematologist, and dentist to ensure that the patient is receiving a well-planned comprehensive treatment [24]. This is essential to ensure the patient is comfortable with their healthcare team so their condition can be managed before it worsens into sickle cell crisis.

Education and spreading awareness of the importance of daily oral health care, as well as encouragement of patients to maintain regular dental check-ups and dental cleanings, is essential. Thus, conducting oral health promotions and screening programs for individuals with SCD is of utmost importance [24].

#### 3.2.2. Strategies to Manage Dental Anxiety

The physical, emotional, and social disabilities from life-long medical and dental issues reinforces dental anxiety over painful procedures, such as tooth extractions, and contributes to avoidance of dental visits [26, 27]. Dental anxiety can be multifactorial and proper evaluation is crucial to identify root causes [26, 28]. Recognizing the etiology and severity of anxiety can help the dental provider better formulate a plan to ensure increased compliance with recommendations.

Evaluation of anxiety can be performed during the initial appointment. Providers can ask patients about their feelings regarding procedures, anesthesia, and sounds along with past dental experience. This can help to inform providers of the patient's level of anxiety prior to dental procedures, so measures can be taken to alleviate their anxiety and make the visit as comfortable as possible [24, 29].

It is important to communicate with the patient regarding the optimal time for their appointment. In general, short morning appointments are recommended.

Pharmacological pain management methods are advised for the mildly anxious patient, which can be achieved by the use of anxiolytics and sedatives, such as midazolam or diazepam [24, 25]. Use of nitrous oxide gas, alone or in combination with a sedative, is also found to be an effective approach for management of dental anxiety in mild-to-moderate cases [30].

In the case of the highly anxious patient requiring extensive multiple dental or surgical procedures, general anesthesia is the most recommended approach [24].

Nonpharmacological pain management strategies include the use of relaxation strategies such as imagery, deep breathing, and distraction. In addition, finding ways to improve the comfort of the environment (e.g., playing music) is another way to help patients feel more relaxed and thus reduce their pain [30].

#### 3.2.3. Restorative Management

Many factors contribute to caries prevalence in SCD, including salivary buffering capacity, salivary flow, improper oral hygiene, systemic conditions, socioeconomic status, and medications [31]. It is important that more proactive measures and a strategic approach are taken to prevent caries and disease spread.

- Caries diagnosis: early detection of caries is the key to prevention. Thus, regular visits to a dentist, at least every 6 months, are recommended for early detection and prevention of dental caries [32].
- Oral hygiene: patient and community education to increase awareness of appropriate oral care is of utmost importance. This includes an emphasis on removing dental plaque daily by brushing twice a day, daily flossing, and use of oral rinses. It is important that the correct brushing techniques are explained and demonstrated for the most effective and efficient results [33].
- Protective methods: regular use of fluoride containing products such as toothpaste, oral mouthwash, fluoride varnish, and calcium phosphate agents can help prevent caries and reverse the oral microflora environment. Pit and fissure sealants, antibacterial, and antimicrobial are other important protective agents [24].
- Diet consultation: high sugar dietary content is a common and well-known etiology of dental caries. Informing patients about the relationship between diet and oral health and helping them reduce sugar content is a helpful way to prevent dental caries [34]. Use of sugar substitute products such as xylitol, which has anticariogenic properties, should be introduced. Consultation with a dietician can help patients understand their individual nutritional needs to determine the appropriate amount and frequency of sugar intake [35]. In addition to the amount of sugar intake, the frequency with which teeth are exposed to sugary substances contributes to poor oral health and reducing such frequency is crucial in preventing dental caries.
- Proactive dental caries treatment: in addition to early diagnosis of dental caries, early intervention is imperative to maintain good oral health. Dental caries can progress aggressively; thus, direct and indirect restorative procedures should be completed in a timely manner to prevent further deterioration of the dentition [34].

#### 3.2.4. Dental Implants

Dental implants are a widely acceptable procedure to replace single or multiple missing teeth. Dental implants are not contraindicated in sickle cell patients; however, it is very crucial to understand this disorder and its clinical physiology to avoid any complications. Due to various clinical manifestations of sickle cell disease, such as osteonecrosis of bone where the blood supply to the jaw is compromised due to clotting in the blood vessels, can cause failure of dental implants [36]. Nevertheless, with meticulous understanding of the nature of the disease, severity of the condition and previous response to procedures can help to plan successful surgery with minimal postoperative complications [37]. Additionally, complete blood count (CBC) and radiographs should be done as a part of the treatment plan. Depending on the CBC results, the patient may need a blood transfusion before or after the surgery to reduce the sickle cell concentration in the blood. In the case where patient may need a blood transfusion, implant surgery should be carried out in the hospital-based setting, with collaborative participation of hematologist and primary care physician [29]. The application of the immediate installation technique has the advantage of achieving satisfactory results with a high success rate. The use of this technique reduces the number of surgical interventions and shortens the time between tooth extraction and permanent installation of the prosthesis, eventually avoiding the process of bone resorption, thereby leading to the preservation of alveolar ridge in terms of proportion, size, and width [38].

#### 3.2.5. Orthodontic Management

Apart from the other orodental manifestations, certain cephalometric changes are characteristic in SCD patients [39]. Orthodontic treatments for the sickle cell disease patient are strictly elective as these patients may have malocclusions or skeletal abnormalities, so their correction can improvise the child's self-esteem.

Some of the common malocclusion features in SCD, including incisal crowding, overjet, open bite, and posterior open bite, are distinctive [40]. Additionally, inclination towards a class II molar relationship, delayed tooth eruption, and increased crowding in the lower anterior region is prominent in children with SCD [41]. It is highly suggested that patients with SCD get orthodontic treatment at the earliest appropriate opportunity to avert problems associated with malocclusions and avoid other complications later in life. Timely orthodontic treatment can help improve quality of life [36]. Orthodontic treatment basically moves teeth through remodeled bone or changes growth patterns by repositioning the lower jaw. However, the disease process of sickle cell disease may compromise the outcome of the planned treatment (van Venrooy and Proffit, 1985) and therefore, treatment ought to be monitored closely, especially during a crisis. Also, orthodontic appliances should be designed with great caution to prevent irritation of soft tissues.

#### 3.2.6. Infection Management

Patients with SCD are at higher risk than the general population of infection, including dental infection [24]. There are several factors contributing to this. Contributing risk factors for various dental and periodontal infections include daily smoking, older age, and lack of daily dental flossing. One of the best ways to prevent dental infection is the early detection and elimination of periodontal and dental sources of infection. Dental infection can impact systematic health through various pathways [42]. Dental infection may also trigger or aggravate sickle cell crises. Thus, all oral infections must be treated aggressively at the local and systematic levels. They must be treated with suitable antimicrobial agents such as antibiotics and rinses [43]. In the case of the severe infection, hospitalization is recommended for administration of intravenous antibiotics, fluids, pain control, and monitoring. Prophylactic antibiotic is recommended for invasive and extensive surgical procedures to prevent systemic infections, vaso-occlusive crisis, and osteomyelitis [12].

However, during sickle cell crisis only acute infections or trauma should be treated, delaying elective procedures until the crisis is resolved.

## 4. Conclusion

This paper broadly describes the oral manifestations of SCD and provides recommendations for better management and understanding of the underlying etiology of such complications. Patients with SCD are often misjudged and, due to lack of knowledge and guidelines, dental providers are not able to provide adequate care. This paper attempts to highlight the essential measures to provide better dental care. It is important that a collaborative approach is adopted with the help of hematologist, dentist, and primary care physician. Early detection, intervention, and prevention are important for improving oral health care in patients with SCD. More research is encouraged to provide evidence for which treatment modalities are most effective.

## Figures and Tables

**Table 1:** Database search strategies.

Database	Keywords	Results
Google Scholar	“Sickle cell” (“Oral Health” OR “dental”)	23,000
Embase	Population—Sickle cell	35
	Intervention—Oral Health	
	Comparison—none	
	Outcome—none	
	“sickle cell anemia”/mj AND “health”/mj	
PubMed	“anemia, sickle cell”[MeSH Terms] AND (“oral health” [MeSH Terms] OR “dental health services” [MeSH Terms])	69
PubMed	“anemia, sickle cell”[MeSH Terms] AND (“oral manifestations” [MeSH Terms]	13

**Table 2:** Challenges in oral management for individuals with sickle cell disease.

Oral health care	(i) Patients with SCD have chronic overall health problems; their hematological disorder becomes their main priority and oral health becomes secondary, increasing their risk for dental caries.	(i) Preventive dental therapy is the best approach for SCD patients (Rada et al., 1987).
	(ii) Patients with SCD are often seen only for emergency appointments when they have severe mouth pain; thus, most of their dental disease is diagnosed during this visit.	(ii) Excellent oral health can reduce the possibility of oral diseases.
	(iii) Lack of regular dental visits and comprehensive care deteriorates their condition significantly, which further demoralizes and demotivates the patient to see a dentist for regular preventive care.	(iii) Incorporate home fluoride treatment (Rouse and Hays, 1979)
		(iv) Incorporate routine dental check-ups (Rouse and Hays, 1979).
Complaint of pain “without any cause”	(i) Patients with SCD often presented clinically with facial and dental pain without an obvious etiology. This makes it difficult for the provider to properly diagnose the reason for that pain [1, 2].	(i) Dentist should perform thorough medical history.
	(ii) Patients are often perceived by health care practitioners as “drug seekers,” which results in delayed effective pain relief often resulting in under treatment that can prolong suffering and result in repeat emergency visits.	(ii) Pale mucosa, delayed eruption of teeth, hypoplasia of teeth, and radiographic changes are common oral signs in SCD patients (Cox and Soni, 1984).
		(iii) Consult a physician before treating the SCD patient.
		(iv) Use acetaminophen for pain as salicylates causes acidosis.
		(v) Regular use of narcotics to alleviate pain should be avoided to prevent drug addiction.
Severe anxiety for dental procedures	SCD patients have severe anxiety towards the oral care. This is mainly due to the severe pain that is experienced on the facial region including maxillary and mandibular bone. Due to this unresolved pain, patients restrict their visit dentist since they are very nervous and uneasy with the overall dental experience.	(i) Oral sedation helped to decrease preoperative anxiety level (Malamed, 1985).
		(ii) Cullen (1982) proposed chloral hydrate or Valium as a premedication for anxiety.
		(iii) Dental appointment can be scheduled during morning time for a short visit (Primley et al., 1982).
Infections	People with SCD have an increased risk of developing certain infections including pneumonia, blood stream infections, meningitis, and bone infections. Early in life, sickled cells can clog blood vessels in the spleen, leading to damage and increased susceptibility to infection.	(i) Antibiotic therapy is recommended for infections and all efforts should be incorporated to prevent acidosis and dehydration (DeBaun and Galadanci) [3].

## References

[1] U.S. National Library of Medicine (2020). Sickle Cell Disease: Sickle Cell Anemia. https://medlineplus.gov/sicklecelldisease.html.

[2] Center for Disease Control and Prevention (2019). Data & statistics on sickle cell disease. https://www.cdc.gov/ncbddd/sicklecell/data.html.

[3] DeBaun M. R., Galadanci N. A. Sickle cell disease in sub-saharan Africa. https://www.uptodate.com/contents/sickle-cell-disease-in-sub-saharan-africa.

[4] Kato G. J., Piel F. B., Reid C. D. (2018). Sickle cell disease. *Nature Reviews Disease Primers*.

[5] World Health Organization (2021). *Sickle Cell Disease*.

[6] Notaloneinsicklecell.com (2021). NotAloneInSickleCell. https://www.notaloneinsicklecell.com/Global-Impact-Of-SCD/.

[7] Piel F. B., Hay S. I., Gupta S., Weatherall D. J., Williams T. N. (2013). Global burden of sickle cell anaemia in children under FIVE, 2010–2050: modelling based on DEMOGRAPHICS, excess mortality, and interventions. *PLoS Medicine*.

[9] Smith H. B., Mcdonald D. K., Miller R. I. (1987). Dental management of patients with sickle cell disorders. *The Journal of the American Dental Association*.

[10] Kawar N., Alrayyes S., Aljewari H. (2018). Sickle cell disease: an overview of orofacial and dental manifestations. *Disease-a-Month*.

[11] Soni N. N. (1966). Microradiographic study of dental tissues in sickle-cell anaemia. *Archives of Oral Biology*.

[12] Little J. W., Miller C. S., Rhodus N. L. (2018). *Little and Falaces Dental Management of the Medically Compromised Patient*.

[13] Pascoe L., Seow W. K. (1994). Enamel hypoplasia and dental caries in Australian aboriginal children: prevalence and correlation between the two diseases. *Pediatric Dentistry*.

[14] Li Y., Navia J. M., Bian J. Y. (1996). Caries experience in deciduous dentition of rural Chinese children 3–5 years old in relation to the presence or absence of enamel hypoplasia. *Caries Research*.

[15] Hong L., Levy S. M., Warren J. J., Broffitt B. (2009). Association between enamel hypoplasia and dental caries in primary second molars: a cohort study. *Caries Research*.

[16] Fukuda J. T., Sonis A. L., Platt O. S., Kurth S. (2005). Acquisition of mutans streptococci and caries prevalence in pediatric sickle cell anemia patients receiving long-term antibiotic therapy. *Pediatric Dentistry*.

[17] Demirbas Kaya A., Aktener B. O., Unsal C. (2004). Pulpal necrosis with sickle cell anaemia. *International Endodontic Journal*.

[18] Andrews C. H., England M. C., Kemp W. B. (1983). Sickle cell anemia: an etiological factor in pulpal necrosis. *Journal of Endodontics*.

[19] Konotey-Ahulc F. I. D. (1972). Mental-nerve neuropathy: a complication of sickle-cell crisis. *The Lancet*.

[20] Friedlander A. H., Genser L., Swerdloff M. (1980). Mental nerve neuropathy: a complication of sickle-cell crisis. *Oral Surgery, Oral Medicine, Oral Pathology*.

[21] Saito N., Nadgir R. N., Flower E. N., Sakai O. (2010). Clinical and radiologic manifestations of sickle cell disease in the head and neck. *RadioGraphics*.

[22] O’Rourke C., Mitropoulos C. (1990). Orofacial pain in patients with sickle cell disease. *British Dental Journal*.

[23] Javed F., Correa F. O. B., Almas K., Nooh N., Romanos G. E., Al-Hezaimi K. (2013). Orofacial manifestations in patients with sickle cell disease. *The American Journal of the Medical Sciences*.

[24] Sams D. R., Thornton J. B., Amamoo P. A. (1990). Managing the dental patient with sickle cell anemia: a review of the literature. *Pediatric Dentistry*.

[25] Kawar N., Alrayyes S., Yang B., Aljewari H. (2018). Oral health management considerations for patients with sickle cell disease. *Disease-a-Month*.

[26] Laurence B., Haywood C., Lanzkron S. (2013). Dental infections increase the likelihood of hospital admission among adult patients with sickle cell disease. *Community Dental Health*.

[27] Beaton L., Freeman R., Humphris G. (2014). Why are people afraid of the dentist? observations and explanations. *Medical Principles and Practice*.

[28] Appukuttan D. (2016). Strategies to manage patients with dental anxiety and dental phobia: literature review. *Clinical, Cosmetic and Investigational Dentistry*.

[29] Moerman N., van Dam F. S. A. M., Muller M. J., Oosting H. (1996). The amsterdam preoperative anxiety and information scale (APAIS). *Anesthesia & Analgesia*.

[30] Aboursheid T., Albaroudi O., Alahdab F. (2019). Inhaled nitric oxide for treating pain crises in people with sickle cell disease. *Cochrane Database of Systematic Reviews*.

[31] Brandão C. F., Oliveira V. M. B., Santos A. R. R. M. (2018). Association between sickle cell disease and the oral health condition of children and adolescents. *BMC Oral Health*.

[32] Laurence B., George D., Woods D. (2006). The association between sickle cell disease and dental caries in African Americans. *Special Care in Dentistry*.

[33] Lee Y. (2013). Diagnosis and prevention strategies for dental caries. *Journal of Lifestyle Medicine*.

[34] Acharya S. (2015). Oral and dental considerations in management of sickle cell anemia. *International Journal of Clinical Pediatric Dentistry*.

[35] Mulimani P., Ballas S. K., Abas A. B., Karanth L. (2019). Treatment of dental complications in sickle cell disease. *Cochrane Database of Systematic Reviews*.

[36] Understanding dental implant complications if you have sickle cell anemia. http://abbey-ltd.com/2016/11/01/understanding-dental-implant-complications-if-you-have-sickle-cell-anemia/.

[37] Hewson I. D., Daly J., Hallett K. B. (2011). Consensus statement by hospital based dentists providing dental treatment for patients with inherited bleeding disorders. *Australian Dental Journal*.

[38] Gusmini M. A. D. S., De Sa A. C., Feng C., Arany S. (2021). Predictors of dental complications post‐dental treatment in patients with sickle cell disease. *Clinical and Experimental Dental Research*.

[39] Pithon M. M. (2011). Orthodontic treatment in a patient with sickle cell anemia. *American Journal of Orthodontics and Dentofacial Orthopedics*.

[40] Nazir M., Basyouni A., Almasoud N., Al-Khalifa K., Al-Jandan B., Al Sulaiman O. (2018). Malocclusion and craniofacial characteristics in Saudi adolescents with sickle cell disease. *Saudi Journal of Medicine and Medical Sciences*.

[41] Pashine A., Shetty R. M., Shetty S. Y., Gadekar T. (2019). Craniofacial and occlusal features of children with sickle cell disease compared to normal standards: a clinical and radiographic study of 50 paediatric patients. *European Archives of Paediatric Dentistry*.

[42] Laurence B., Haywood C., Lanzkron S. (2013). Dental infections increase the likelihood of hospital admissions among adult patients with sickle cell disease. *Community Dental Health*.

[43] da Fonseca M. A., Oueis H. S., Casamassimo P. S. (2007). Sickle cell anemia: a review for the pediatric dentist. *Pediatric Dentistry*.

